# An introduction to gender affirming healthcare: What the family physician needs to know

**DOI:** 10.4102/safp.v65i1.5770

**Published:** 2023-07-31

**Authors:** Madeleine Muller, Elma de Vries, Anastacia Tomson, Christine McLachlan

**Affiliations:** 1 Department of Family Medicine and Rural Health, Faculty of Health Sciences, Walter Sisulu University, East London, South Africa; 2 Department of Family Medicine, Cecilia Makiwane Hospital, East London, South Africa; 3 School of Medicine, Faculty of Health Sciences, Nelson Mandela University, Gqeberha, South Africa; 4 My Family GP, Cape Town, South Africa; 5 Shemah Koleinu, Cape Town, South Africa; 6 Department of Psychology, Faculty of Social Sciences, University of South Africa, Pretoria, South Africa

**Keywords:** gender affirming healthcare, transgender, key populations, patient centred care, gender diverse, access to care, hormone therapy

## Abstract

Gender affirming healthcare (GAHC) is a relatively new field in primary health care that describes a range of gender affirming practices, including hormone therapy, for transgender and gender diverse (TGD) people. In 2019, gender affirming hormones were approved by South African National Essential Medicine List Committee (NEMLC) for tertiary-level care, and in October 2021 the Southern Africa HIV Clinicians Society published a GAHC guideline for South Africa. Unfortunately, TGD people still experience discrimination and stigmatisation in healthcare facilities in South Africa, leading to poor access to care and higher health risks with poorer outcomes. Gender affirming care in the primary health care clinic can improve access to health care, with improved provision of preventative services. This article defines key transgender concepts, describes the informed consent process and outlines the initiation and monitoring of both feminising and masculinising hormone treatment for TGD people. Staff at healthcare facilities need to receive training on gender affirming practices and how to ensure a safe environment for TGD clients.

## Introduction

In October 2021, the Southern Africa HIV Clinicians Society published a much-needed Gender Affirming Healthcare guideline (GAHC) for South Africa.^1,2^ Gender Affirming Healthcare guideline is a relatively new field in medicine and the guideline hopes to address the significant gaps in knowledge and skills of healthcare providers in providing GAHC to transgender and gender diverse (TGD) people. In addition, the recent Ritshidze report outlines the ongoing poor attitudes of healthcare workers as reported by TGD people, negatively affecting access to care.^3^ This continuing professional development article touches on some of the principles of GAHC and introduces gender affirming hormone therapy. For an in-depth understanding, see the expanded GAHC guideline.^2^

In 2018, the World Health Organization (WHO) published the International Classification of Disease (ICD-11), in which ‘gender identity disorder’ was removed from the section of mental health disorders, reflecting the ‘current knowledge that trans-related and gender diverse identities are not conditions of mental ill-health’.^4^ Despite that it is known that transgender identity is an established and recognised phenomenon, widespread stigmatisation and discrimination have a huge impact on the health and well-being of this key population and specific medical guidelines are therefore needed. Essential to the management of TGD individuals is gender affirmation, which Sevelius defines as an ‘interpersonal, interactive process whereby a person receives social recognition and affirmation for their gender identity and expression’.^5^ Gender affirmation does not follow a fixed formula, and differs from individual to individual, but may include a variety of processes, for example, social actions such as using the correct pronouns, as well as hormone therapy and gender affirming surgery.

In McWhinney and Freeman’s family medicine principles outlined in 2016,^6^ the family physician is cautioned to be aware of their own subjective milieu and its influence on their response to their patients. Healthcare workers themselves are part of cultural or religious constructs, which may have taboos around sexual orientation and gender identity. As family physicians we need to be able to best manage the patient in front of us and provide the medical care our patient needs, regardless of personal views. The family medicine principles provide an excellent framework for quality care of our transgender patients, considering the individual’s needs as well as the context of our patient.

## Transgender 101: Key concepts

For most clinicians, GAHC includes many new and possibly unfamiliar concepts.

*Transgender* is a term that describes a person who does not identify (wholly or partially) with their sex assigned at birth. A *transgender woman* (TGW) is someone who was assigned male at birth, but who identifies as a woman and usually uses the pronouns she and her. A transgender man (TGM) is someone who was assigned female at birth, but who identifies as a man, usually using pronouns he and him.

*Gender diverse* is a general term and relates to a person whose gender identity or gender expression does not conform to socially defined male or female gender norms and includes those who identify as gender fluid or non-binary. Gender fluid or non-binary people often use the singular they and them pronouns, which is commonly used when referring to gender neutral persons such as ‘There is somebody at the door. Please ask *them* to come in’.

It is important to distinguish gender identity from sexual orientation, which refers to the gender of the person that one is attracted to, or the absence of attraction.

It is often asked why there seems to be an increasing number of people who identify as TGD. The increased prevalence of left-handedness in the 20th century provides a possible explanation. In 1939, only 2.1% of the population was considered left-handed, but this had increased to 12% in 1972 as acceptance grew.^7^ Although TGD individuals have always formed part of human society, cultural and religious taboos have meant that they have not been visible in many communities. In 2023, there are still eight countries where being TGD is illegal and carries the death penalty.^8^ As TGD identities are becoming more accepted, an increasing number of people are willing to speak out. Ten years ago a TGD patient in your consulting room would have been a rare event. As acceptance increases, so will prevalence and we need to be prepared.

## Preparing your facility for gender affirming healthcare

A study performed by Luvuno et al.^9^ in KwaZulu-Natal in 2019 highlighted the lack of facilities, resources and targeted programmes catering for TGD people. Access is further compounded by negative attitudes from staff. The researchers recommend that training of healthcare workers will improve the healthcare provision for this population.

With gender affirming care available within a primary care setting, TGD people are able to access preventative care options and pre-exposure prophylaxis and receive mental health support and appropriate disease screening. This is in line with the National Strategic plan on HIV, TB and STI of 2017–2022,^10^ where transgender people are recognised as a key population with poor access to preventative services, largely because of stigmatisation by healthcare professionals.

Over and above hormone therapy, much needs to happen to ensure that TGD individuals feel safe to visit a healthcare facility. The first step will be the training of all staff including the security guard at the front gate, the filing clerks, the nurses and the doctors as well as the wider multidisciplinary team consisting of social workers, speech therapists and occupational therapists. Gender affirmation starts with the healthcare worker affirming and accepting the patient’s gender identity. All staff in the facility need to be sensitive to using the client’s true name (the name with which a person identifies, which may be different from a person’s given or legal name) and correct pronouns, and the correct use of gender terminology.

Management needs to be engaged to ensure that consulting rooms afford privacy and confidentiality and to ensure there is a safe bathroom the patient can use, for example, designating one of the toilet spaces as a gender-neutral toilet. Consider a young 16-year-old trans boy, assigned female at birth, needing to visit a public bathroom, and his fear of confrontation (e.g., by women if he uses a female bathroom) or the possible risk of gender-based violence when using a male bathroom. The expanded GAHC guideline delves into these issues in depth, including considerations around admission of a TGD person to a hospital.^2^

## Hormone therapy

The GAHC moved away from a gatekeeping model, where the doctor ‘decided’ whether the patient was ‘trans’ enough to qualify for hormone therapy. Tomson recommends informed consent, with the patient taking responsibility for the decision regarding gender affirmation treatment.^11^ The duty of the clinician is to provide appropriate and comprehensive information to help the individual make that decision. Its focus is to embrace a collaborative process whereby a TGD person is empowered. This article will focus only on informed consent in the adult patient for hormone therapy.

Informed consent involves the following:

- Confirm the client’s desire to start hormone therapy and ask about their goals, hopes, and concerns. It is important to manage expectations. Bodily changes will be determined by the client’s genetic predisposition and this needs to be discussed in depth.
- Cover the timelines of the different bodily changes and their reversibility (Table 1 and Table 2)
- Discuss the different options, side-effects and potential complications of hormone therapy
- Examine fertility and reproductive options
- Analyse possible impact on mental health and the psychosocial impact of transitioning.

**TABLE 1:** Timeline and reversibility of feminising hormone therapy.

Effect	Time from initiation to onset	Time from initiation to maximum effect	Reversible
Body fat distribution	3–6 months	2–3 years	Yes
Decreased muscle mass and strength	3–6 months	1–2 years	Yes
Skin softening	3–6 months	Unknown/variable	Yes
Change in sexual desire	1–3 months	3–6 months	Yes
Decreased erections	1–3 months	3–6 months	Yes
Breast growth	3–6 months	4 years	Yes
Decreased sperm production	Unknown/variable	> 3 years	Possibly
Decreased terminal hair growth	6–12 months	> 3 years	Yes
Scalp hair	Variable	Unknown/variable	Yes
Voice changes	None	n/a	n/a

*Source*: Tomson A, McLachlan C, Wattrus C, et al. Southern African HIV Clinicians Society gender-affirming healthcare guideline for South Africa – Expanded version [homepage on the Internet]. October 2021 [cited 2023 Mar 29]. Available from: https://sahivsoc.org/Files/SAHCS%20GAHC%20guidelines-expanded%20version_Oct%202021(3).pdf

n/a, not applicable.

**TABLE 2:** Timeline and reversibility of masculinising hormone therapy.

Effect	Time from initiation to onset	Time from initiation to maximum effect	Reversible
Skin oiliness acne	1–6 months	1–2 years	Yes
Facial and body hair growth	6–12 months	4–5 years	No
Scalp hair loss	6–12 months	Unknown	No
Increased muscle mass and strength	6–12 months	2–5 years	Yes
Fat distribution	1–6 months	2–5 years	Yes
Cessation of menses	2–6 months	n/a	Possibly
Clitoral hypertrophy	3–6 months	1–2 years	No
Vaginal atrophy	3–6 months	1–2 years	Yes
Deepening of voice	6–12 months	1–2 years	No

*Source*: Tomson A, McLachlan C, Wattrus C, et al. Southern African HIV Clinicians Society gender-affirming healthcare guideline for South Africa – Expanded version [homepage on the Internet]. October 2021 [cited 2023 Mar 29]. Available from: https://sahivsoc.org/Files/SAHCS%20GAHC%20guidelines-expanded%20version_Oct%202021(3).pdf

n/a, not applicable.

The guideline includes a Client Information and Informed consent form for both masculinising and feminising treatment in its appendices.^1,2^

Informed consent can be taken by any prescribing clinician confident in the process. Where prescribers do not have enough time or capacity or where the individual is particularly vulnerable, it is advisable to involve a psychologist, clinical social worker or mental health practitioner who has had training and experience in the field of GAHC.

### The ins and outs of hormone therapy

The hormones used in GAHC have been shown to be safe and effective and in 2019 gender affirming hormones were approved by the South African National Essential Medicine List Committee (NEMLC) for tertiary-level of care.^12^ This has been an important step to provide access to gender affirming hormone therapy, and more work is needed to ensure such treatment be included in the primary and secondary hospital EML. Many transgender programmes are managed by NGOs within a primary health care setting.

Before starting therapy, the clinician must perform a baseline assessment, which includes a thorough history and screening for relevant conditions including liver disease, kidney disease, heart disease, hypertension, venous thromboembolism (VTE) disease, malignancy, dyslipidaemia, diabetes and other endocrine or metabolic conditions.

A physical examination is usually performed, but it is not necessary to do a genital or breast examination unless there is a specific indication. Baseline blood tests will be determined by the prescribed hormones such as the importance of a baseline haemoglobin in patients starting on testosterone. In Table 3, there are suggested baseline screening tests, depending on the clinical setting and the risk profile of the patient. Depending on availability and acceptability, the clinician will now choose the appropriate hormone regime.

**TABLE 3:** Suggested baseline screening prior to hormone therapy.

Suggested baseline screening and investigations	Resource-constrained setting
Full blood count	Haemoglobin
Urea, electrolytes, creatinine	Creatinine
Liver function test panel	Alanine transaminase
Fasting plasma glucose	Finger-prick glucose
Fasting lipogram	Total cholesterol

*Source*: Tomson A, McLachlan C, Wattrus C, et al. Southern African HIV Clinicians Society gender-affirming healthcare guideline for South Africa – Expanded version [homepage on the Internet]. October 2021 [cited 2023 Mar 29]. Available from: https://sahivsoc.org/Files/SAHCS%20GAHC%20guidelines-expanded%20version_Oct%202021(3).pdf

Note: HIV, hepatitis B and syphillis screen if indicated. Pregnancy test if a client assigned female at birth is started on masculinising therapy.

### Feminising hormone therapy

The cornerstone of feminising therapy (Tables 4 and 5) is the administration of exogenous oestrogen. The preferred method is parenteral administration, as it is associated with lower risk of adverse events and is not subject to first-pass metabolism in the liver, but unfortunately parenteral oestrogens are not readily available in the public sector. When using an oral oestrogen, it is often necessary to combine it with an anti-androgen such as spironolactone, to ensure the suppression of testosterone.

**TABLE 4:** Feminising hormone therapy: Oestrogens.

Medication	Dosing	Notes
Estradiol valerate (IM or SC)	Start: 6 mg/week Increase by 2 mg at a time Maintenance: 6 mg – 10 mg/week Max dose: 20 mg/week	Preferred route Low relative risk of VTE Anti-androgen not needed
Estradiol valerate (oral) 1 mg or 2 mg tablets, for example, Estrofem	Start 2 mg per day Increase by 2 mg at a time Maintenance: 6 mg/day – 8 mg/day Max 8 mg/day	Often requires coadministration of anti-androgen Moderate relative risk of VTE
Estradiol (patch) From 25 mcg to 100 mcg patches, for example, Estradot/Climera/Evorel	Start 50 mcg – 100 mcg twice a week Increase by 100 mcg at a time Maintenance 300 mcg – 400 mcg twice weekly Maximum: 400 mcg twice weekly	Low relative risk of VTE Anti-androgen not needed Possible side effects (rare) – allergy to adhesive/skin irritation
Conjugated equine oestrogen (oral) 0.3 mg, 0.625 mg, 1.25 mg tabs, for example, Premarin	Start 0.625 mg – 1.25 mg/day Increase by 0.625 mg – 1.25 mg at a time Usual maintenance dose 1.25 mg – 2.5 mg daily Max dose: 5 mg/day	Often requires anti-androgen Moderate relative risk of VTE Only use when no other preparations available

*Source*: Tomson A, McLachlan C, Wattrus C, et al. Southern African HIV Clinicians Society gender-affirming healthcare guideline for South Africa – Expanded version [homepage on the Internet]. October 2021 [cited 2023 Mar 29]. Available from: https://sahivsoc.org/Files/SAHCS%20GAHC%20guidelines-expanded%20version_Oct%202021(3).pdf

IM, intramuscular; SC, subcutaneous; VTE, venous thromboembolism.

**TABLE 5:** Feminising hormone therapy: Antiandrogen.

Medication	Dosing	Notes
Spironolactone (oral) 25 mg or 100 mg tabs	Start: 25 mg/day Increase by 25 mg at a time Maintenance: 50 mg Max dose: 200 mg	Risk of hyperkalaemia – requires potassium monitoring Possible side effects: diarrhoea, abdominal cramping, nausea, vomiting, headache, dizziness
Cyproterone acetate (oral) 10 mg or 50 mg or 100 mg tabs	Start 10 mg/day – 12.5 mg/day Increase by 5 mg – 6.25 mg at a time Maintenance: 10 mg/day – 25 mg/day Max dose: 25 mg/day	Potent anti-androgen – low doses should be sufficient Possible side effects: sweating, agitation, fluid retention at high doses
Bicalutamide (oral) 50 mg or 150 mg tabs	Start 25 mg twice a week Increase by 25 mg twice a week Maintenance: 25 mg – 50 mg daily Max dose: 50 mg/day	Preferred antiandrogen – less risk of neuro-steroid depletion Possible side effects: constipation, back pain and fatigue

*Source*: Tomson A, McLachlan C, Wattrus C, et al. Southern African HIV Clinicians Society gender-affirming healthcare guideline for South Africa – Expanded version [homepage on the Internet]. October 2021 [cited 2023 Mar 29]. Available from: https://sahivsoc.org/Files/SAHCS%20GAHC%20guidelines-expanded%20version_Oct%202021(3).pdf

Oestrogen therapy is safe, and is commonly used, for example, in contraception. The most significant risk is that of VTE, with a small risk in young healthy patients (2.3/1000 patient years) and a higher risk in those aged > 35 years, smokers and those who are obese.^13^ Side effects are similar to using oestrogen in other settings: migraine, nausea, mood changes and changes to libido and the sexual response cycle. As with hormone replacement therapy, caution is needed in oestrogen sensitive malignancies, cardiovascular disease and cerebrovascular disease.

### Masculinising hormone therapy

For masculinising therapy (Table 6), only testosterone is needed. It is usually administered as an intramuscular or subcutaneous injection, with topical transdermal preparations available in private.

**TABLE 6:** Masculinising hormone therapy.

Medication	Dosing	Notes
Testosterone cypionate 100 mg/mL (IM or SC) Short acting, for example, Depo Testosterone, Pfizer	Start: 50 mg (0.5 mL) per week Increase by 10 mg (0.1 mL) at a time Maintenance: 50 mg – 80 mg (0.5 mL – 0.8 mL) weekly or 100 mg – 200 mg every 2 weeks. Max dose: 100 mg (1 mL) weekly or 200 mg every 2 weeks	More affordable May take a sample of testosterone midway between dosages (at peak) – target safe upper limit of target range for men
Testosterone undecanoate (IM) 1000 mg/4 mL Long acting, for example, Nebido	Start: 1000 mg every 10–12 weeks Increase the frequency, rather than the dosing Maintenance: 1000 mg every 10–12 weeks Max dose: 1000 mg	More expensive than short-acting Achieving the correct dose can be difficult with long dosing intervals Take sample for testosterone measurement at trough, target the lower level of reference range
Topical testosterone, for example, Androgel	Start dose: 1 sachet (5 mL) applied topically daily Increase by 1 mL at a time Maintenance: varies Max dose: limited by body surface for application	Only available from compounding pharmacies

*Source*: Tomson A, McLachlan C, Wattrus C, et al. Southern African HIV Clinicians Society gender-affirming healthcare guideline for South Africa – Expanded version [homepage on the Internet]. October 2021 [cited 2023 Mar 29]. Available from: https://sahivsoc.org/Files/SAHCS%20GAHC%20guidelines-expanded%20version_Oct%202021(3).pdf

IM, intramuscular; SC, subcutaneous.

Testosterone is safe to use and results in a similar risk profile to that of a cisgender man. Clients should be monitored for dyslipidaemias, hypertension and polycythaemia and testosterone must be avoided in pregnancy. Over and above these risks possible side effects include acne, amenorrhea (usually desired), loss of fertility and mood changes.

### Monitoring of patients on hormone therapy

Patients should be reviewed 1 month after starting hormone treatment, at three-monthly intervals in the first year and twice a year thereafter. Dose titration and maintenance is primarily driven by client experience and can be adjusted accordingly. Measurement of serum hormone levels is not necessary, but can be used in a private setting where resources are available. Ongoing management of expectations and provision of a comprehensive package of care is essential.

## Conclusion

The expanded GAHC guideline covers in depth the informed consent process including children and adolescents; aspects around opportunistic health screening and prevention; non-medical gender-affirming practices such as binding, tucking, padding and packing; surgery; voice and communication and includes a section on institutions such as schools and the workplace. To do a full online training on the GAHC guidelines sign up for the free course offered by the HIV clinician Society of South Africa (launched 31 March 2023) https://sahivsoc.org/Subheader/Index/gender-affirming-healthcare-training-course.

Gender affirming healthcare is safe and effective and can be best practised in a primary health care setting, ensuring TGD people have access to preventative health care services and mental health and social support. Family physicians and primary health care staff need to be sensitised to the use of gender affirming practices, and trained in the provision of gender affirming hormone treatment. As stated by Audre Lorde, ‘It is not our differences that divide us. It is our inability to recognize, accept, and celebrate those differences’.^14^ We hope this article inspires clinicians to learn more about providing quality care for TGD people.
